# Numerical simulation of MHD double diffusive natural convection and entropy generation in a wavy enclosure filled with nanofluid with discrete heating

**DOI:** 10.1016/j.heliyon.2019.e02496

**Published:** 2019-09-24

**Authors:** Rujda Parveen, T.R. Mahapatra

**Affiliations:** Department of Mathematics, Visva-Bharati (A Central University), Institute of Science, Santiniketan - 731 235, West-Bengal, India

**Keywords:** Electromagnetism, Mechanical engineering, Nonlinear physics, Thermodynamics, Natural convection, Center heater, Double diffusive, Magnetohydrodynamics, Nanofluid, Entropy generation, Wavy walled enclosure

## Abstract

A numerical investigation of entropy generation, heat and mass transfer is performed on steady double diffusive natural convection of water-based Al_2_O_3_ nanofluid within a wavy-walled cavity with a center heater under the influence of an uniform vertical magnetic field. The top horizontal wavy wall, left and right vertical walls of the enclosure are kept at low temperature and concentration of $T_{c}$ and $c_{c}$ whereas central part of the bottom horizontal wall is maintained at high temperature and concentration of $T_{h}$ and $c_{h}$ and the remaining part is kept adiabatic where temperature and concentration gradient are taken as zero. The Bi-CGStab method and Tri-diagonal algorithm are used to solve the governing equations. The study has been performed for several relevant parameters such as Rayleigh number ($10^{3}\leq Ra\leq 10^{5}$), Hartmann number (0≤Ha≤60), buoyancy ratio number (−2≤N≤2), volume fraction of nanoparticles (0.0≤ϕ≤0.2) and different undulation number of the upper wavy wall (*n*). The Prandtl number and Lewis number are kept fixed at Pr=6.2 and Le=2. The effect of these parameters are revealed in terms of streamlines, isotherms, isoconcentrations, entropy generation, average Nusselt number and Sherwood number. Results indicate that heat and mass transfer rate augment as Rayleigh number and volume fraction of nanoparticles increase and are found to drop with the increase in Hartmann number and buoyancy ratio.

## 1 Introduction

Fluid dynamics induced by the combination of temperature and concentration gradients is called double-diffusive convection. Double diffusive natural convection heat transfer has fundamental aspects in modern life. Some applications of double diffusive natural convection occur in engineering fields such as natural gas storage tanks, drying processes, solar ponds, material processing etc. as well as in scientific fields such as astrophysics, biology and chemical processes, geosciences etc. In most of these applications, numerous enclosures of various shapes (including rectangular, triangular, trapezoidal, rhomboidal, sinusoidal or ellipsoidal) have been considered to analyze the heat and mass transfer effects. Lee and Hyun [1] investigated the double-diffusive convection in a rectangular enclosure. They reported that Nusselt number reduces monotonically as the buoyancy ratio rises from a small value. The analysis of double-diffusive convection in vertical enclosures for different aspect ratios and Lewis numbers have been studied by Ghorayeb and Mojtabi [2]. Mahapatra et al. [3] analyzed the effect of buoyancy ratio on double-diffusive mixed convection with uniform and non-uniform heating of walls.

In literature, convection in nanofluids has been extensively investigated by many researchers, due to the thermal conductivity enhancing feature of the nanofluid ([4], [5], [6], [7], [8]). Some applications of nanofluid to enhance heat transfer performance includes heat exchangers, solar energy, cooling of electronic devices equipped with nanofluids, food processing etc. ([9], [10]). The book written by Das et al. [11] contains many studies on natural convection in nanofluids. Esfahani and Bordbar [12] simulated double-diffusive natural convection heat transfer enhancement in a square enclosure filled with various nanofluids. They discussed the impact of the volume fraction of nanoparticle and Lewis number on Nusselt number and Sherwood number. Parvin et al. [13] numerically analyzed the double diffusive natural convection of water-Al_2_O_3_ nanofluid in a partially heated enclosure. They established that the heat transfer rate is most effective in case of highest Rayleigh number. Nasrin and Alim [14] numerically investigated laminar double diffusive convection in a prism shaped solar collector using water-CuO nanofluid. Chen et al. [15] numerically investigated entropy generation on double diffusive natural convection in a rectangular enclosure filled with nanofluid.

The impact of the magnetohydrodynamics on convective heat transfer in an enclosure has been carried out to determine the effects of the magnetic field on the heat transfer either in conduction or convection mode. Ghasemi et al. [16] carried out natural convection in a differentially heated square enclosure filled with Al_2_O_3_-water nanofluid in presence of magnetic field. They conveyed that the heat transfer rate increases and decreases with the increase of Rayleigh number and Hartmann number respectively. Teamah [17] studied double diffusive flow in a rectangular cavity in the presence of magnetic field and inner heat source. They reported that the fluid circulation and heat and mass transfer rate within the enclosure reduces with the increase in magnetic field effect. Teamah and Shehata [18] carried out MHD double diffusive natural convection in a trapezoidal enclosure with various inclination angles. They found that rate of heat and mass transfer decreases with the increase in inclination angle and Hartmann number. Rahman et al. [19] investigated MHD double-diffusive mixed convection in a horizontal channel with an open cavity. Mahapatra et al. [20] presented numerical study of double-diffusive natural convection in a trapezoidal enclosure filled with nanofluid under the influence of magnetic field.

Some engineering applications that are associated to partial heating and cooling zones such as solar energy collection, effective cooling of electronic components, prevention of subsoil water pollution etc. have become increasingly important with the fast growth of electronic technology. In such practical applications complete effective walls are not taken into account for heat and mass transfer. Also the corresponding location of the hot and cold wall regions plays an important role in optimizing heat and mass transfer rate in the enclosure. Natural convection is the only favorable mode of cooling the heat source in many applications. Therefore, the study of convective heat and mass transfer in the enclosures having partially active thermal walls is required in order to achieve a better understanding of these applications. Calcagni et al. [21] numerically and experimentally studied the convective heat transfer in a square enclosure having discrete heater placed on the lower wall. They obtained that for high Rayleigh number, heat transfer increases with an increase in dimension of the heat source. Oueslati et al. [22] have numerically investigated double-diffusive natural convection with entropy generation in an enclosure partially heated and salted from the left vertical sidewall. Kandaswamy et al. [23] numerically investigated magnetoconvection in a square enclosure having partially active vertical walls. They estimated that the heat transfer rate for the middle-middle thermally active locations was maximum as compared to top-bottom thermally active locations. Also the average Nusselt number enhances with rise in Grashof number but decreases with increase in Hartmann number. Natural convective heat transfer of different types of nanofluid filled rectangular enclosure with partially heated left vertical wall was studied by Oztop and Abu-Nada [24]. They obtained the results that by increasing the value of Rayleigh number, volume fraction of nanoparticles and heater size enhances the heat transfer rate. Aminossadati and Ghasemi [25] evaluated that the heat transfer augments with increasing the length of heater located at the bottom wall of a square enclosure filled with nanofluid. Chamkha and Al-Naser [26] performed MHD double-diffusive convection along the left and right walls of the enclosure having constant heat and mass fluxes. Cho [27] discussed entropy generation of natural convection in a square cavity having partially-heated wavy surface and filled with nanofluid. Teamah [28] made numerical simulation of MHD double diffusive natural convection in a rectangular enclosure filled with nanofluid. They found that the fluid circulation, heat and mass transfer reduces in presence of magnetic field.

Natural convection heat transfer in corrugated or wavy enclosures is gaining attention of most of the researchers for enhancing the efficiency of heat and mass transfer. Over the last few years, natural convection in wavy enclosures have been carried out by various researchers ([29], [30], [31], [32], [33], [34]). Although, double-diffusive natural convection has obtained less attention in complex enclosures, it has significant applications in various engineering fields such as solidification in material processing, chemical engineering, food industries, cement manufacturing. The combined process of heat and mass transfer was analyzed by Rathish Kumar and Krishna Murthy [35] from a wavy vertical surface immersed in a fluid-saturated semi-infinite porous medium. Hussain [36] analyzed numerically heatline and entropy generation during double diffusive MHD natural convection in a tilted sinusoidal corrugated porous enclosure. Gholizadeh et al. [37] examined double diffusive natural convection in a partially heated trapezoidal enclosure. They carried out the work for different position of the thermal active wall and different inclination angle of the side walls.

After literature survey, we found that researchers have examined natural convection and entropy generation in various enclosures. Sometime they have considered double diffusive natural convection and entropy generation in a wavy enclosure filled with nanofluid and sometime they have considered MHD natural convection in a wavy enclosure with discrete heating filled with nanofluid. But, all these characteristics at the same time have not been considered so far. Hence in the current work, we aim to investigate MHD double diffusive natural convection and entropy generation of aluminium-water nanofluid in a wavy enclosure having discrete heater placed on the lower wall. We incorporate nanofluid medium inside the enclosure as it greatly affect the heat transfer rate.

## 2 Model

**Figure 1:**
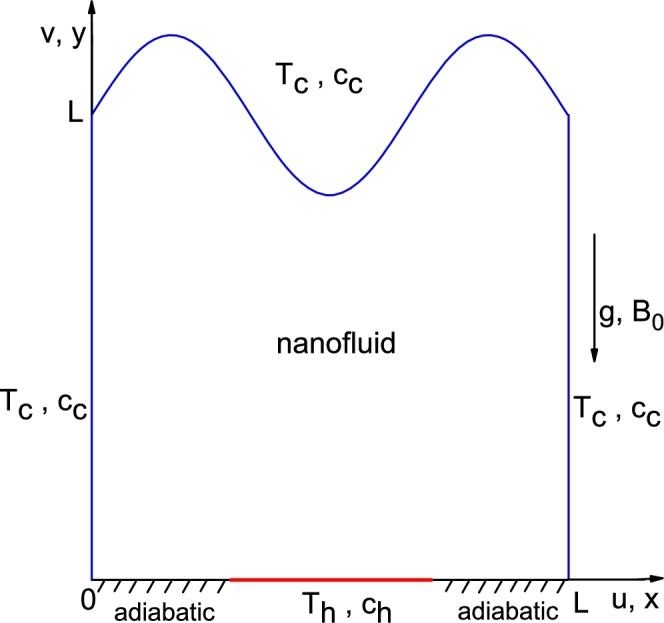
Schematic representation of boundary conditions.

A schematic diagram of a wavy-walled enclosure with cartesian coordinates (x,y) and velocity components (u,v) is shown in Fig. 1. The width and height of the enclosure is L and the fluid inside is water-based nanofluid including Al_2_O_3_ nanoparticles. It is assumed that the top wavy wall and the vertical walls are maintained at low temperature $\left(T_{c}\right)$ and concentration $\left(c_{c}\right)$. The temperature and concentration of the central part of the bottom wall are $T_{h}(>T_{c})$ and $c_{h}(>c_{c})$, respectively, whereas zero gradient of temperature and concentration are maintained at the rest part of the lower wall. Distance of the heat and concentration source from both the vertical walls is exactly the same. A uniform magnetic field with constant magnitude $B_{0}$ and the gravitational force are applied vertically normal to the horizontal wall. It is considered that the upper wavy wall of the enclosure is defined by the relation:

$$f(x)=L+Asin(n\pi \frac{x}{L})$$

where *A* is the amplitude of the sinusoidal wall and *n* is the undulation number of the upper wall. The symbols used here are defined in Table 1.

**Table 1:** Nomenclature

*x*, *y*	distance along x and y coordinate, m
*X*, *Y*	dimensionless distance along x and y coordinate
*u*, *v*	x and y components of velocity
*U*, *V*	x and y components of dimensionless velocity
*T*	temperature of the fluid, K
*p*	pressure, Pa
*P*	dimensionless pressure
*C*_*p*_	Specific heat at constant pressure, *Jkg*^−1^*K*^−1^
*D*	Mass Diffusivity, *m*^2^*s*^−1^
*a*	amplitude of the wavy wall
*g*	acceleration due to gravity, *ms*^−2^
*k*	thermal conductivity,*Wm*^−1^*K*^−1^
*c*	dimensional solute concentration (*kgm*^−3^)
*C*	dimensionless solute concentration
*ν*	kinematic viscosity, *m*^2^*s*^−1^
*ρ*	density, kg *m*^−3^
*θ*	dimensionless temperature
*α*	thermal diffusivity, *m*^2^*s*^−1^
*β*_*s*_	volumetric coefficient of thermal expansion, *K*^−1^
*β*_*T*_	volumetric coefficient of solutal expansion, *m*^3^*kg*^−1^
*μ*	dynamic viscosity, kg *m*^−1^*s*^−1^
*σ*	Electrical conductivity, *A*^2^*s*^3^*m*^−3^*kg*^−1^
*ψ*	dimensionless stream function
*ξ*	transformed horizontal coordinate
*η*	transformed vertical coordinate
*c*	cold
*h*	hot
*nf*	nanofluid

## 3 Theory/Calculation

### 3.1 Thermo-physical properties of nanofluid

The flow is considered to be laminar, steady and incompressible and the thermo-physical properties of base fluid and nano-sized particles are tabulated in Table 2.

**Table 2:** Thermophysical properties of the base fluid (pure water) and nanoparticle.

Physical properties	Pure water	*Al*_2_*O*_3_
*C*_*p*_ (J kg^−1^ K^−1^)	4179	765
*ρ* (kg m^−3^)	997.1	3970
*k* (W m^−1^ K^−1^)	0.613	40
*α* × 10^7^ (m^2^ s^−1^)	1.47	131.7
*β* (K^−1^)	21 × 10^−5^	0.85 × 10^−5^
*μ* (kg m^−1^ s^−1^)	0.001003	-

The effective density $\left(\rho_{nf}\right)$, specific heat ${\left(\rho C_{p}\right)}_{nf}$, thermal expansion coefficient ${\left(\rho \beta_{T}\right)}_{nf}$ of the nanofluid, according to [25], are:

$$(\rho_{nf})=(1-\phi )\rho_{f}+\phi \rho_{s},\;{\left(\rho C_{p}\right)}_{nf}=(1-\phi ){\left(\rho C_{p}\right)}_{f}+\phi {\left(\rho C_{p}\right)}_{s}\;\text{and }{\left(\rho \beta_{T}\right)}_{nf}=(1-\phi ){\left(\rho \beta_{T}\right)}_{f}+\phi {\left(\rho \beta_{T}\right)}_{s}.$$

Effective dynamic viscosity, $\mu_{nf}$ and the effective thermal conductivity, $k_{nf}$ of the nanofluid which are obtained from Brinkman model [38] and Maxwell's model [39] respectively, are introduced as:

$$\mu_{nf}=\mu_{f}{\left(1-\phi \right)}^{-2.5}\;\text{and}\;k_{nf}=k_{f}\left[\frac{k_{s}+2k_{f}-2\phi (k_{f}-k_{s})}{k_{s}+2k_{f}+\phi (k_{f}-k_{s})}\right].$$

Finally, the thermal diffusivity and electrical conductivity of the nanofluid are defined respectively as:

$$\alpha_{nf}=\frac{k_{nf}}{{\left(\rho C_{p}\right)}_{nf}}\;\text{and}\;\sigma_{nf}=\sigma_{f}\left(1+\frac{3(\zeta -1)\phi }{(\zeta +2)-(\zeta -1)\phi }\right).$$

Here *ϕ* represents the volume fraction of the nanoparticle and $\zeta =\sigma_{s}/\sigma_{f}$. The subscript *f* and *s* are used to refer base fluid and solid particle, respectively.

### 3.2 Governing equations

The equations which govern the two dimensional steady double-diffusive natural convection flow of an electrically conducting incompressible nanofluid are given in dimensional form by

Continuity equation:

(1) $$\frac{\partial u}{\partial x}+\frac{\partial v}{\partial y}=0.$$

Momentum conservation equations:

(2) $$u\frac{\partial u}{\partial x}+v\frac{\partial u}{\partial y}=-\frac{1}{\rho_{nf}}\frac{\partial p}{\partial x}+\nu_{nf}\left[\frac{\partial^{2}u}{\partial x^{2}}+\frac{\partial^{2}u}{\partial y^{2}}\right]-\frac{\sigma_{nf}\;B_{0}^{2}\;u}{\rho_{nf}},$$

(3) $$u\frac{\partial v}{\partial x}+v\frac{\partial v}{\partial y}=-\frac{1}{\rho_{nf}}\frac{\partial p}{\partial y}+\nu_{nf}\left[\frac{\partial^{2}v}{\partial x^{2}}+\frac{\partial^{2}v}{\partial y^{2}}\right]+\frac{g{\left(\rho \beta_{T}\right)}_{nf}}{\rho_{nf}}(T-T_{0})-\frac{g{\left(\rho \beta_{S}\right)}_{nf}}{\rho_{nf}}(c-c_{0}).$$

Energy equation:

(4) $$u\frac{\partial T}{\partial x}+v\frac{\partial T}{\partial y}=\alpha_{nf}\left[\frac{\partial^{2}T}{\partial x^{2}}+\frac{\partial^{2}T}{\partial y^{2}}\right].$$

Concentration equation:

(5) $$u\frac{\partial c}{\partial x}+v\frac{\partial c}{\partial y}=D\left[\frac{\partial^{2}c}{\partial x^{2}}+\frac{\partial^{2}c}{\partial y^{2}}\right].$$

The particular boundary conditions of the problem in dimensional form are given by

u=0, v=0, $T=T_{c}$ and $c=c_{c}$ for 0≤y≤L and x=0,L,
u=0, v=0, $T=T_{c}$ and $c=c_{c}$ for 0≤x≤L and y=L,
u=0, v=0, $T=T_{h}$ and $c=c_{h}$ for 0.3L≤x≤0.7L and y=0,
u=0, v=0, $\frac{\partial T}{\partial y}=0$ and $\frac{\partial c}{\partial y}=0$ for 0≤x≤0.3L, 0.7L≤x≤L and y=0.

Following transformation of variables are used to convert the system (1)-(5) into non-dimensional form:

$$X=\frac{x}{L},\;Y=\frac{y}{L},\;U=\frac{uL}{\alpha_{f}},\;V=\frac{vL}{\alpha_{f}},\;P=\frac{pL^{2}}{\rho_{nf}\alpha_{f}^{2}},\;\theta =\frac{T-T_{0}}{T_{h}-T_{c}},\;C=\frac{c-c_{0}}{c_{h}-c_{c}}$$

where $T_{0}$ and $C_{0}$ are the mean temperature and concentration of heated and cooled walls defined by,

$$T_{0}=\frac{T_{h}+T_{c}}{2}\;\text{and}\;C_{0}=\frac{C_{h}+C_{c}}{2}.$$

The resulting non-dimensional continuity, momentum, energy and concentration equations can be written as:

(6) $$\frac{\partial U}{\partial X}+\frac{\partial V}{\partial Y}=0,$$

(7) $$U\frac{\partial U}{\partial X}+V\frac{\partial U}{\partial Y}=-\frac{\partial P}{\partial X}+\frac{\mu_{nf}}{\rho_{nf}\alpha_{f}}\left[\frac{\partial^{2}U}{\partial X^{2}}+\frac{\partial^{2}U}{\partial Y^{2}}\right]-\frac{\sigma_{nf}\rho_{f}}{\sigma_{f}\rho_{nf}}Ha^{2}UPr,$$

(8) $$U\frac{\partial V}{\partial X}+V\frac{\partial V}{\partial Y}=-\frac{\partial P}{\partial Y}+\frac{\mu_{nf}}{\rho_{nf}\alpha_{f}}\left[\frac{\partial^{2}V}{\partial X^{2}}+\frac{\partial^{2}V}{\partial Y^{2}}\right]+\frac{{\left(\rho \beta_{s}\right)}_{nf}}{\rho_{nf}\beta_{f}}\;RaPr(\theta -NC),$$

(9) $$U\frac{\partial \theta }{\partial X}+V\frac{\partial \theta }{\partial Y}=\frac{\alpha_{nf}}{\alpha_{f}}\left[\frac{\partial^{2}\theta }{\partial X^{2}}+\frac{\partial^{2}\theta }{\partial Y^{2}}\right],$$

(10) $$U\frac{\partial C}{\partial X}+V\frac{\partial C}{\partial Y}=\frac{1}{Le}\left[\frac{\partial^{2}C}{\partial X^{2}}+\frac{\partial^{2}C}{\partial Y^{2}}\right],$$

where the Prandtl number $Pr=\frac{\nu_{f}}{\alpha_{f}}$, Lewis number $Le=\frac{\alpha_{f}}{D}$, Rayleigh number $Ra=\frac{g\;\beta_{f}(T_{h}-T_{c})L^{3}}{\nu_{f}\alpha_{f}}$, Hartmann number $Ha=\sqrt{\frac{\sigma_{f}}{\mu_{f}}}\;B_{0}L$, and Buoyancy ratio $N=\frac{{\left(\rho \beta_{s}\right)}_{nf}(c_{h}-c_{c})}{{\left(\rho \beta_{T}\right)}_{nf}(T_{h}-T_{c})}$.

The upper sinusoidal wall in non-dimensional form can be written as:

$$\frac{f(x)}{L}=1+\frac{A}{L}sin(n\pi \frac{x}{L}),\text{i.e.}\;\;F(X)=1+asin(n\pi X),$$

where a=A/L(=0.2) is the amplitude (*A*) of the upper wall taken as 0.2*L*.

The initial and boundary conditions in non-dimensional form are:

U=0, V=0 and θ=−0.5=C, for 0≤Y≤1 and X=0, 1,
U=0, V=0 and θ=−0.5=C, for 0≤X≤1 and Y=1,
U=0, V=0 and θ=0.5=C, for 0.3≤X≤0.7 and Y=0,
U=0, V=0 and $\frac{\partial \theta }{\partial Y}=0=\frac{\partial C}{\partial Y}$, for 0≤X≤0.3, 0.7≤X≤1 and Y=0.

### 3.3 Nusselt number and Sherwood number

The local and average heat and mass transfer are given in dimensionless terms by the Nusselt and Sherwood numbers, respectively.

The local Nusselt number (Nu) and local Sherwood number (Sh) along the discrete heat source at the lower wall are defined by

(11) $$Nu=-\frac{k_{nf}}{k_{f}}\frac{\partial \theta }{\partial Y}\;\;\text{and}\;\;Sh=-\frac{\partial C}{\partial Y}.$$

The average Nusselt number $\left(Nu_{avg}\right)$ and average Sherwood number $\left(Sh_{avg}\right)$ are obtained by integrating local Nusselt number (Nu) and local Sherwood number (Sh) along the discrete heat source.

(12) $$Nu_{avg}=\int_{0.3}^{0.7}NudX\;\;\text{and}\;\;Sh_{avg}=\int_{0.3}^{0.7}ShdX.$$

To evaluate eq. (12), a Simpson's $\frac{1}{3}$rd rule of integration is implemented.

### 3.4 Entropy generation

The dimensionless forms of the local entropy generation expression using the dimensionless quantities are given by:

$$S_{\theta }=\frac{k_{nf}}{k_{f}}\left[{\left(\frac{\partial \theta }{\partial X}\right)}^{2}+{\left(\frac{\partial \theta }{\partial Y}\right)}^{2}\right],S_{\psi }=\lambda_{1}\frac{\mu_{nf}}{\mu_{f}}\left[2\left({\left(\frac{\partial U}{\partial X}\right)}^{2}+{\left(\frac{\partial V}{\partial Y}\right)}^{2}\right)+{\left(\frac{\partial U}{\partial Y}+\frac{\partial V}{\partial X}\right)}^{2}\right],S_{m}=\lambda_{1}\frac{\sigma_{nf}}{\sigma_{f}}Ha^{2}U^{2},S_{d}=\lambda_{2}\left[{\left(\frac{\partial C}{\partial X}\right)}^{2}+{\left(\frac{\partial C}{\partial Y}\right)}^{2}\right]+\lambda_{3}\left[\left(\frac{\partial C}{\partial X}\right)\left(\frac{\partial \theta }{\partial X}\right)+\left(\frac{\partial C}{\partial Y}\right)\left(\frac{\partial \theta }{\partial Y}\right)\right],$$

where

$$\lambda_{1}=\frac{\mu_{f}T_{0}}{k_{f}}\left(\frac{{\alpha_{f}}^{2}}{L^{2}{\left(T_{h}-T_{c}\right)}^{2}}\right),\lambda_{2}=\frac{RDT_{0}}{k_{f}C_{0}}{\left(\frac{c_{h}-c_{c}}{T_{h}-T_{c}}\right)}^{2},\lambda_{3}=\frac{RD}{k_{f}}\left(\frac{c_{h}-c_{c}}{T_{h}-T_{c}}\right),$$

are called as irreversibility distribution ratio. Here, *R* is the gas constant.

The average entropy generation due to heat transfer $\left(S_{\theta ,avg}\right)$, fluid friction $\left(S_{\psi ,avg}\right)$, magnetic field $\left(S_{m,avg}\right)$ and due to diffusion $\left(S_{d,avg}\right)$ are obtained by integrating the local entropy generation by the system volume

$$S_{\theta ,avg}=\int_{V}S_{\theta }dV,\;\;S_{\psi ,avg}=\int_{V}S_{\psi }dV,S_{m,avg}=\int_{V}S_{m}dV,\;\;S_{d,avg}=\int_{V}S_{d}dV.S_{t,avg}=S_{\theta ,avg}+S_{\psi ,avg}+S_{m,avg}+S_{d,avg}.$$

## 4 Methodology

### 4.1 Numerical method

The stream function (*ψ*) and vorticity (*ω*) in non-dimensional form are given by:

(13) $$U=\frac{\partial \psi }{\partial Y},\;\;V=-\frac{\partial \psi }{\partial X}\;\;\text{and}\;\;\omega =\frac{\partial V}{\partial X}-\frac{\partial U}{\partial Y},$$

which gives a single equation

(14) $$\frac{\partial^{2}\psi }{\partial X^{2}}+\frac{\partial^{2}\psi }{\partial Y^{2}}=-\omega .$$

Using eq. (13) and eliminating pressure term from eq. (7) and (8), we get

(15) $$Q_{1}\left(\frac{\partial^{2}\omega }{\partial X^{2}}+\frac{\partial^{2}\omega }{\partial Y^{2}}\right)-\left(U\frac{\partial \omega }{\partial X}+V\frac{\partial \omega }{\partial Y}\right)\;+Q_{2}RaPr\left(\frac{\partial \theta }{\partial X}-N\frac{\partial C}{\partial X}\right)+Q_{3}Ha^{2}Pr\frac{\partial U}{\partial Y}=0$$

where $Q_{1}=\frac{\mu_{nf}}{\rho_{nf}\alpha_{f}}$, $Q_{2}=\frac{{\left(\rho \beta \right)}_{nf}}{\rho_{nf}\beta_{f}}$ and $Q_{3}=\frac{\sigma_{nf}\rho_{f}}{\sigma_{f}\rho_{nf}}$.

**Figure 2:**
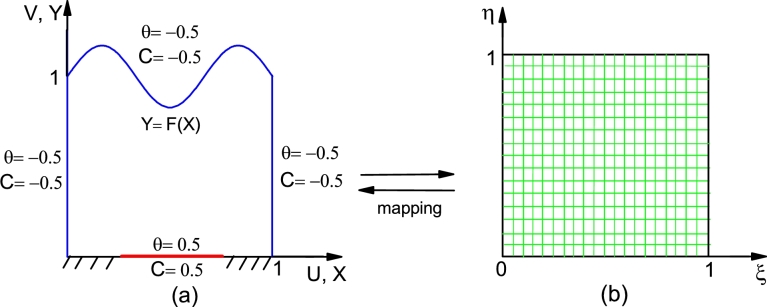
Mapping of physical domain (a) to computational domain (b).

The presence of wavy upper wall make it difficult to impose the wavy boundary on rectangular grids. Thus, transformation is required to convert the irregular physical domain (X,Y) into a regular (square) computational domain (ξ,η), see Fig. 2. In this study we have considered the following algebraic relations

(16) ξ=Xη=Y/(1+asinnπX).

The upper sinusoidal boundary Y=F(X) is transformed into the straight line η=1 using the above transformation.

The equations can be evaluated in ξ−η domain using the following relationship

$$\left(\begin{array}{cc} \frac{\partial \xi }{\partial X} & \frac{\partial \xi }{\partial Y}\\ \frac{\partial \eta }{\partial X} & \frac{\partial \eta }{\partial Y} \end{array}\right)=\frac{1}{J}\left(\begin{array}{cc} \frac{\partial Y}{\partial \eta } & -\frac{\partial X}{\partial \eta }\\ -\frac{\partial Y}{\partial \xi } & \frac{\partial X}{\partial \xi } \end{array}\right)$$

where,

$$J=\frac{\partial (X,Y)}{\partial (\xi ,\eta )}=\left|\begin{array}{cc} \frac{\partial X}{\partial \xi } & \frac{\partial X}{\partial \eta }\\ \frac{\partial Y}{\partial \xi } & \frac{\partial Y}{\partial \eta } \end{array}\right|$$

is the Jacobian of the transformation.

Taking into account transformation (16) and chain rule of differentiation, the governing eqs. (9), (10), (12) and (15) are respectively transformed as:

(17) $$a_{3}\frac{\partial^{2}\theta }{\partial \xi^{2}}+e_{3}\frac{\partial^{2}\theta }{\partial \xi \partial \eta }+b_{3}\frac{\partial^{2}\theta }{\partial \eta^{2}}+c_{3}\frac{\partial \theta }{\partial \xi }+d_{3}\frac{\partial \theta }{\partial \eta }=0$$

(18) $$a_{4}\frac{\partial^{2}C}{\partial \xi^{2}}+e_{4}\frac{\partial^{2}C}{\partial \xi \partial \eta }+b_{4}\frac{\partial^{2}C}{\partial \eta^{2}}+c_{4}\frac{\partial C}{\partial \xi }+d_{4}\frac{\partial C}{\partial \eta }=0$$

(19) $$a_{1}\frac{\partial^{2}\psi }{\partial \xi^{2}}+e_{1}\frac{\partial^{2}\psi }{\partial \xi \partial \eta }+b_{1}\frac{\partial^{2}\psi }{\partial \eta^{2}}+c_{1}\frac{\partial \psi }{\partial \xi }+d_{1}\frac{\partial \psi }{\partial \eta }=-\omega$$

(20) $$a_{2}\frac{\partial^{2}\omega }{\partial \xi^{2}}+e_{2}\frac{\partial^{2}\omega }{\partial \xi \partial \eta }+b_{2}\frac{\partial^{2}\omega }{\partial \eta^{2}}+c_{2}\frac{\partial \omega }{\partial \xi }+d_{2}\frac{\partial \omega }{\partial \eta }+Q_{3}Ha^{2}Pr\left(\frac{1}{J}\frac{\partial U}{\partial \eta }\right)\;+Q_{2}RaPr\left[\left(\frac{\partial \theta }{\partial \xi }+\frac{e_{1}}{2}\frac{\partial \theta }{\partial \eta }\right)-N\left(\frac{\partial C}{\partial \xi }+\frac{e_{1}}{2}\frac{\partial C}{\partial \eta }\right)\right]=0$$

where

(21) $$U=\frac{1}{J}\frac{\partial \psi }{\partial \eta }\;\;\text{and}\;\;V=-\frac{\partial \psi }{\partial \xi }-\frac{e_{1}}{2}\frac{\partial \psi }{\partial \eta }.$$

Here,

$$a_{1}=\frac{1}{J^{2}}(x_{\eta }^{2}+y_{\eta }^{2}),\;\;b_{1}\;=\;\frac{1}{J^{2}}(x_{\xi }^{2}+y_{\xi }^{2}),\;\;e_{1}\;=\;\frac{-2}{J^{2}}(y_{\eta }y_{\xi }+x_{\eta }x_{\xi }),c_{1}=\frac{1}{J^{3}}\left[-y_{\eta }((x_{\eta }^{2}+y_{\eta }^{2})x_{\xi \xi }-2(y_{\eta }y_{\xi }+x_{\eta }x_{\xi })x_{\xi \eta }+(x_{\xi }^{2}+y_{\xi }^{2})x_{\eta \eta })\right]+\frac{1}{J^{3}}\left[x_{\eta }((x_{\eta }^{2}+y_{\eta }^{2})y_{\xi \xi }-2(y_{\eta }y_{\xi }+x_{\eta }x_{\xi })y_{\xi \eta }+(x_{\xi }^{2}+y_{\xi }^{2})y_{\eta \eta })\right],d_{1}=\frac{1}{J^{3}}\left[-y_{\xi }((x_{\eta }^{2}+y_{\eta }^{2})x_{\xi \xi }-2(y_{\eta }y_{\xi }+x_{\eta }x_{\xi })x_{\xi \eta }+(x_{\xi }^{2}+y_{\xi }^{2})x_{\eta \eta })\right]+\frac{1}{J^{3}}\left[-x_{\xi }((x_{\eta }^{2}+y_{\eta }^{2})y_{\xi \xi }-2(y_{\eta }y_{\xi }+x_{\eta }x_{\xi })y_{\xi \eta }+(x_{\xi }^{2}+y_{\xi }^{2})y_{\eta \eta })\right],a_{2}=a_{1}Q_{1},\;\;b_{2}\;=\;b_{1}Q_{1},\;\;e_{2}\;=\;e_{1}Q_{1},c_{2}=-\frac{1}{J}(uy_{\eta }-vx_{\eta })+c_{1}Q_{1},\;\;d_{2}\;=\;-\frac{1}{J}(-uy_{\xi }+vx_{\xi })+d_{1}Q_{1}\;a_{3}=a_{1}\frac{\alpha_{nf}}{\alpha_{f}},\;\;b_{3}\;=\;b_{1}\frac{\alpha_{nf}}{\alpha_{f}},\;\;e_{3}\;=\;e_{1}\frac{\alpha_{nf}}{\alpha_{f}},c_{3}=-\frac{1}{J}(uy_{\eta }-vx_{\eta })+c_{1}\frac{\alpha_{nf}}{\alpha_{f}},\;\;d_{3}\;=\;-\frac{1}{J}(-uy_{\xi }+vx_{\xi })+d_{1}\frac{\alpha_{nf}}{\alpha_{f}}\;a_{4}=a_{1}\frac{1}{Le},\;\;b_{4}\;=\;b_{1}\frac{1}{Le},\;\;e_{4}\;=\;e_{1}\frac{1}{Le},c_{4}=-\frac{1}{J}(uy_{\eta }-vx_{\eta })+c_{1}\frac{1}{Le},\;\;d_{4}\;=\;-\frac{1}{J}(-uy_{\xi }+vx_{\xi })+d_{1}\frac{1}{Le}.$$

Substituting *ω* from eq. (19) and writing in (20), we get the following biharmonic equation in stream function-velocity formulation

(22) a2∂4ψ∂ξ4+2e2∂4ψ∂ξ3∂η+T1∂4ψ∂ξ2∂η2+2e2b1∂4ψ∂ξ∂η3+=0b1b2∂4ψ∂η4+c2∂3ψ∂ξ3+T2∂3ψ∂ξ2∂η+T3∂3ψ∂ξ∂η2+T4∂3ψ∂η3+=0T5∂2ψ∂ξ∂η+T6∂2ψ∂η2+T7∂ψ∂η−Q3Ha2Pr(1J∂U∂η)=0−Q2RaPr[(∂θ∂ξ+e12∂θ∂η)−N(∂C∂ξ+e12∂C∂η)]=0.

Here,

$$T_{1}=a_{2}b_{1}+e_{1}e_{2}+a_{1}b_{2},T_{2}=2a_{2}e_{1\xi }+a_{2}d_{1}+a_{1\xi }e_{2}+e_{1\eta }e_{2}+e_{2}c_{1}+2b_{2}a_{1\eta }+c_{2}e_{1}+d_{2}a_{1},T_{3}=2a_{2}b_{1\xi }+e_{2}e_{1\xi }+e_{2}b_{1\eta }+e_{2}d_{1}+2b_{2}e_{1\eta }+b_{2}c_{1}+c_{2}b_{1}+d_{2}e_{1},T_{4}=e_{2}b_{1\xi }+2b_{1\eta }b_{2}+b_{2}d_{1}+d_{2}b_{1},T_{5}=e_{1\xi \xi }a_{2}+2d_{1\xi }a_{2}+e_{1\xi \eta }e_{2}+c_{1\xi }e_{2}+d_{1\eta }e_{2}+e_{1\eta \eta }b_{2}+2c_{1\eta }b_{2}+e_{1\xi }c_{2}+c_{2}d_{1}+d_{2}e_{1\eta }+d_{2}c_{1},T_{6}=b_{1\xi \xi }a_{2}+b_{1\xi \eta }e_{2}+d_{1\xi }e_{2}+b_{1\eta \eta }b_{2}+2d_{1\eta }b_{2}+b_{1\xi }c_{2}+b_{1\eta }d_{2}+d_{1}d_{2},T_{7}=d_{1\xi \xi }a_{2}+d_{1\xi \eta }e_{2}+d_{1\eta \eta }b_{2}+d_{1\xi }e_{2}+d_{1\eta }d_{2}.$$

The transformed eqs. (22), (17) and (18) are then discretized using second order central difference scheme and can be written in matrix form as

(23) $$\text{A}\psi =f\left(Ra,Pr,U,V,\frac{\partial \theta }{\partial \xi },\frac{\partial \theta }{\partial \eta },\frac{\partial C}{\partial \xi },\frac{\partial C}{\partial \eta },\frac{\partial \psi }{\partial \xi },\frac{\partial \psi }{\partial \eta }\right)$$

(24) Bθ=0

(25) SC=0

where the coefficient matrix A, B and S are of order *mn* and *ψ* and *f* are *mn*-component vectors for a grid of size m×n.

The discretization of any function Φ (such as ψ,θ,C etc.) having *d* as the step length on a uniform rectangular mesh in the transformed domain, are given as:

$$\frac{\partial \Phi }{\partial \xi }=\frac{1}{2d}(\Phi_{i+1,j}-\Phi_{i-1,j})+O(d^{2}),\;\;\frac{\partial \Phi }{\partial \eta }=\frac{1}{2d}(\Phi_{i,j+1}-\Phi_{i,j-1})+O(d^{2}),\frac{\partial^{2}\Phi }{\partial \xi^{2}}=\frac{1}{d^{2}}(\Phi_{i+1,j}-2\Phi_{i,j}+\Phi_{i-1,j})+O(d^{2}),\frac{\partial^{2}\Phi }{\partial \eta^{2}}=\frac{1}{d^{2}}(\Phi_{i,j+1}-2\Phi_{i,j}+\Phi_{i,j-1})+O(d^{2}),\frac{\partial^{3}\Phi }{\partial \xi^{3}}=\frac{1}{d^{2}}[{\left(\Phi_{\xi }\right)}_{i+1,j}-2{\left(\Phi_{\xi }\right)}_{i,j}+{\left(\Phi_{\xi }\right)}_{i-1,j}]+O(d^{2}),\frac{\partial^{3}\Phi }{\partial \xi^{2}\partial \eta }=\frac{1}{2d^{3}}(2\Phi_{i,j-1}-2\Phi_{i,j+1}-\Phi_{i-1,j-1}-\Phi_{i+1,j-1}+\Phi_{i+1,j+1}+\Phi_{i-1,j+1})+O(d^{2}),\frac{\partial^{3}\Phi }{\partial \xi \partial \eta^{2}}=\frac{1}{2d^{3}}[2\Phi_{i-1,j}-2\Phi_{i+1,j}-\Phi_{i-1,j-1}+\Phi_{i+1,j-1}+\Phi_{i+1,j+1}-\Phi_{i-1,j+1}]+O(d^{2}),\frac{\partial^{3}\Phi }{\partial \eta^{3}}=\frac{1}{d^{2}}[{\left(\Phi_{\eta }\right)}_{i,j+1}-2{\left(\Phi_{\eta }\right)}_{i,j}+{\left(\Phi_{\eta }\right)}_{i,j-1}]+O(d^{2}),$$

$$\frac{\partial^{4}\Phi }{\partial \xi^{4}}=\frac{6}{d^{4}}[d({\left(\Phi_{\xi }\right)}_{i+1,j}-{\left(\Phi_{\xi }\right)}_{i-1,j})-2(\Phi_{i+1,j}-2\Phi_{i,j}+\Phi_{i-1,j})]+O(d^{2}),\frac{\partial^{4}\Phi }{\partial \eta^{4}}=\frac{6}{d^{4}}[d({\left(\Phi_{\eta }\right)}_{i,j+1}-{\left(\Phi_{\eta }\right)}_{i,j-1})-2(\Phi_{i,j+1}-2\Phi_{i,j}+\Phi_{i,j-1})]+O(d^{2}),\frac{\partial^{4}\Phi }{\partial \xi^{3}\partial \eta }=\frac{1}{2d^{3}}[2{\left(\Phi_{\xi }\right)}_{i,j-1}-2{\left(\Phi_{\xi }\right)}_{i,j+1}-{\left(\Phi_{\xi }\right)}_{i-1,j-1}-{\left(\Phi_{\xi }\right)}_{i+1,j-1}+{\left(\Phi_{\xi }\right)}_{i+1,j+1}+{\left(\Phi_{\xi }\right)}_{i-1,j+1}]+O(d^{2}),\frac{\partial^{4}\Phi }{\partial \xi \partial \eta^{3}}=\frac{1}{2d^{3}}[2{\left(\Phi_{\eta }\right)}_{i-1,j}-2{\left(\Phi_{\eta }\right)}_{i+1,j}-{\left(\Phi_{\eta }\right)}_{i-1,j-1}+{\left(\Phi_{\eta }\right)}_{i+1,j-1}+{\left(\Phi_{\eta }\right)}_{i+1,j+1}-{\left(\Phi_{\eta }\right)}_{i-1,j+1}]+O(d^{2}),\frac{\partial^{4}\Phi }{\partial \xi^{2}\partial \eta^{2}}=\frac{1}{d^{4}}[4\Phi_{i,j}-2(\Phi_{i-1,j}+\Phi_{i+1,j}+\Phi_{i,j-1}+\Phi_{i,j+1})+\Phi_{i-1,j-1}+\Phi_{i+1,j-1}+\Phi_{i+1,j+1}+\Phi_{i-1,j+1}]+O(d^{2}).$$

Eqs. (23), (24) and (25) are solved by using Bi-CGStab method. Detailed explanation of the used numerical scheme is presented by [40], [41]. The tri diagonal system

(26) $${\left(\frac{\partial \psi }{\partial \xi }\right)}_{i+1,j}+4{\left(\frac{\partial \psi }{\partial \xi }\right)}_{i,j}+{\left(\frac{\partial \psi }{\partial \xi }\right)}_{i-1,j}=\frac{3}{d}(\psi_{i+1,j}-\psi_{i-1,j}),$$

(27) $${\left(\frac{\partial \psi }{\partial \eta }\right)}_{i,j+1}+4{\left(\frac{\partial \psi }{\partial \eta }\right)}_{i,j}+{\left(\frac{\partial \psi }{\partial \eta }\right)}_{i,j-1}=\frac{3}{d}(\psi_{i,j+1}-\psi_{i,j-1}),$$

which arises from the fourth order finite difference approximation of ∂ψ/∂ξ and ∂ψ/∂η is solved by using Thomas algorithm to get ∂ψ/∂ξ and ∂ψ/∂η. After obtaining ∂ψ/∂ξ and ∂ψ/∂η we compute *U* and *V* from eq. (21).

The convergence criterion is that the difference of values of all the variables between two consecutive iterations is less than $0.5\times 10^{-6}$.

### 4.2 Code validation

**Figure 3:**
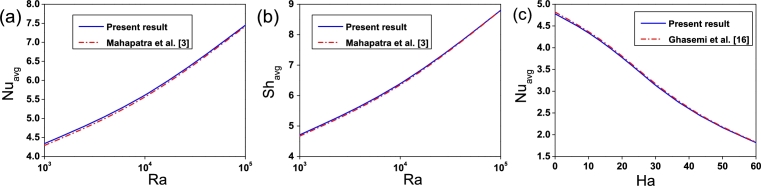
(a), (b) Comparison of present results with Mahapatra et al. [3] and (c) Ghasemi et al. [16].

The present results have been authenticated successfully with the results of Mahapatra et al. [3] in a square enclosure maintained at different temperatures and concentrations and shown in Fig. 3(a) and 3(b). The average Nusselt number and average Sherwood number at the lower wall were compared when Pr=0.7, Le=2.0 and N=−1.0 for various values of Rayleigh number. Fig. 3 (a) and 3(b) illustrate good agreement with the data of the authors. The second test was performed to compare our work with the work of Ghasemi et al. [16] for natural convection in a $Al_{2}O_{3}$-water nanofluid filled square enclosure having adiabatic horizontal walls and isothermal vertical walls maintained at different temperatures at Pr=6.2, ϕ=0.02 and $Ra=10^{5}$. Fig. 3(c) shows a good agreement for different values of Hartmann number, and therefore, we get the confidence of the present numerical code.

### 4.3 Grid independence test

Grid independence study is displayed in Table 3 for different grid sizes 21×21, 41×41, 81×81, 161×161 for (a) $Ra=10^{3}$, Ha=30 and (b) $Ra=10^{5}$, Ha=0, ϕ=0.1 N=−2 and n=3. The result shows insignificant change for grid size of 81×81 and higher. Hence for the present study the grid size of 81×81 is chosen for all computations.

**Table 3:** Grid independence test for |*ψ*|_*max*_ at *ϕ* = 0.1, *N* = −2 and *n* = 3.

Grid size					
*Ra*	*Ha*	21 × 21	41 × 41	81 × 81	161 × 161

10^3^	30	0.0970	0.1072	0.1072	0.1071
10^5^	0	15.0811	15.8212	16.2611	16.2212

## 5 Results & Discussion

In this paper, we have considered Magnetohydrodynamic double-diffusive natural convection of $Al_{2}O_{3}$-water nanofluid in a wavy enclosure. The enclosure is discretely heated and concentrated from lower wall and the rest walls are kept at comparatively less temperature and concentration. The influence of different physical parameters such as Rayleigh number, undulation number, volume fraction of nanoparticles, Hartmann number, buoyancy ratio are examined in terms of streamlines, isotherms, isoconcentrations, entropy generation, average Nusselt number and average Sherwood number.

### 5.1 Effects of Rayleigh number

**Figure 4:**
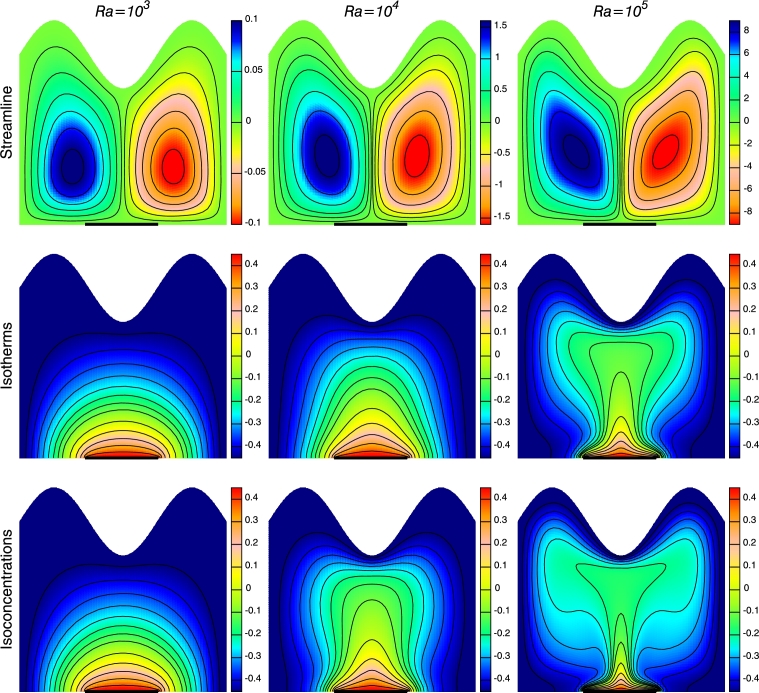
Streamlines, Isotherms and Isoconcentrations for different *Ra* at *Ha* = 30, *N* = −2, *n* = 3 and *ϕ* = 0.1.

Fig. 4 shows the influence of Rayleigh number on streamlines, isotherms and isoconcentrations for three different values of the Rayleigh numbers, viz. $Ra=10^{3}$, 10^4^ and 10^5^ when N=−2, n=3, Ha=30 and ϕ=0.1. Due to the temperature difference, the fluid rises from the middle of the bottom wall and falls along the sides of the cold vertical walls forming counter-rotating vortices within the enclosure. At low $Ra(=10^{3})$, the strength of stream function is weak due to conduction mode of heat transfer. The enhancement in *Ra* escalates the buoyant force and consequently the magnitude of stream function increases, as can be seen from Fig. 4. However, rise in *Ra* does not effect the shape of the circulating vortices but the center of rotation changes from oval to slightly tilted oval and is found to be displaced from bottom to core region of the enclosure. As the heat source is placed in the middle of the lower wall, temperature and concentration patterns are parallel near the discrete heat source at low *Ra* which signifies conduction dominant flow. Maximum heat transfer occurs at the center of the cavity. Significant change in isotherms and isoconcentrations are observed with the enhancement in *Ra*. Isotherm and isoconcentration contours become distorted due to enhanced convection effect at high *Ra* and show similar behavior due to similar energy and mass equation. However, isoconcentration lines are found to be more distorted as compared to isothermal lines resulting in higher mass transfer.

### 5.2 Effects of Hartmann number

**Figure 5:**
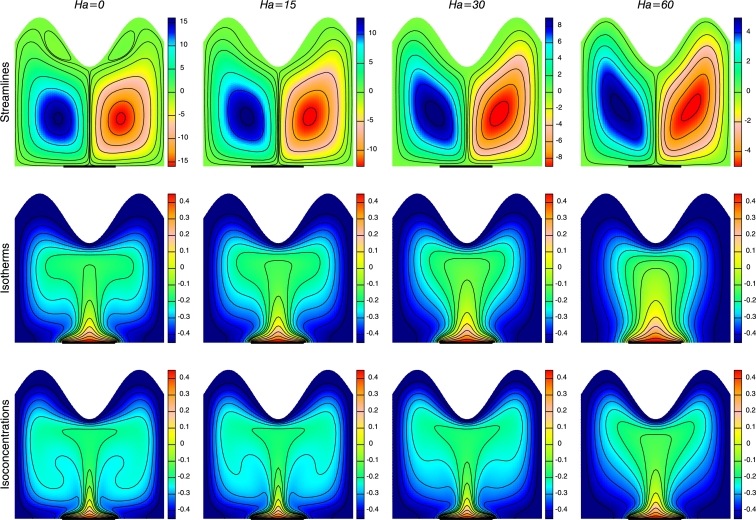
Streamlines, Isotherms and Isoconcentrations for different *Ha* at *Ra* = 10^5^, *N* = −2, *n* = 3 and *ϕ* = 0.1.

Fig. 5 demonstrates the effect of magnetic field on streamlines, isotherms and isoconcentrations considering other parameters fixed. In absence of magnetic field (Ha=0) the value of stream function, $|\psi {|}_{max}=16.26$ which shows stronger convection effect. It is observed that even though the pattern of streamlines are same, the strength of stream function decreases with the augmentation of Lorentz forces due to weak flow under the influence of magnetic field, as can be seen from Fig. 5. The temperature and concentration distribution shows significant effect with rise in *Ha*. The isotherm and isoconcentration contours generate plume like distributions in the interior enclosure in the absence of magnetic field (Ha=0) indicating strong convection effect. Thermal and solutal boundary layer are found to be highly compressed near the walls and empty at the center of the enclosure. The plumes diminishes, becomes parallel and gathered adjacent to the horizontal wall with the increase in Ha(=60) tending to inhibit the convection effect due to presence of Lorentz force. It is observed that the presence of strong magnetic field leads to intensification of conductive mode of heat transfer not convective mode of heat transfer.

### 5.3 Effects of buoyancy ratio

**Figure 6:**
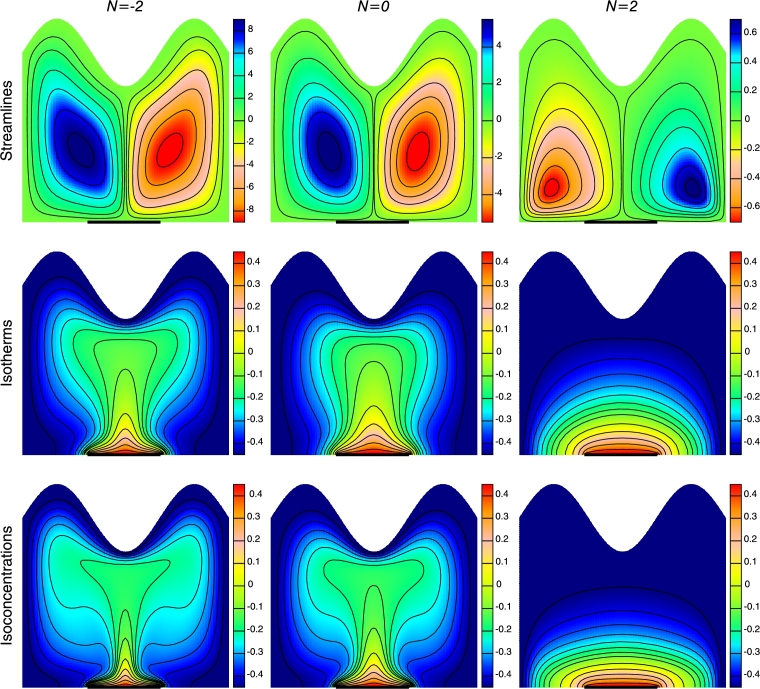
Streamlines, Isotherms and Isoconcentrations for different Buoyancy ratios (*N*) at *Ra* = 10^5^, *Ha* = 30, *n* = 3 and *ϕ* = 0.1.

The impact of buoyancy ratio (*N*) on streamlines, isotherms and isoconcentrations is displayed in Fig. 6. The buoyancy ratio can be defined as the ratio of solutal buoyancy to thermal buoyancy forces and it measures correspondingly the significance of thermal and mass diffusion in the buoyancy-driven flow. It is observed that the flow is effected by the concentration gradient for N=−2, causing domination of solutal buoyancy force over thermal buoyancy force. This tends to increase in flow strength and can be considered as aiding flow as can be seen in Fig. 6. The contours of temperature and concentration are found to be skewed in the core of the enclosure. However, isoconcentration contours are more distorted than isotherm contours signifying higher mass transfer. When the buoyancy ratio increases, taking positive values (N=2), the thermal and solutal buoyancy forces oppose each other and tries to counterbalance each others effect. The fluid then starts to flow in completely reverse direction forming opposing flow that leads to decrease in flow strength. It is found that the flow is more intense in the middle of the enclosure. Also the temperature and concentration pattern becomes parallel to the horizontal wall resulting in conduction mode of heat transfer. The isoconcentration contours show similar behavior as that of isotherm due to similar energy and mass transfer equation.

### 5.4 Effects of nanoparticle volume fraction

**Figure 7:**
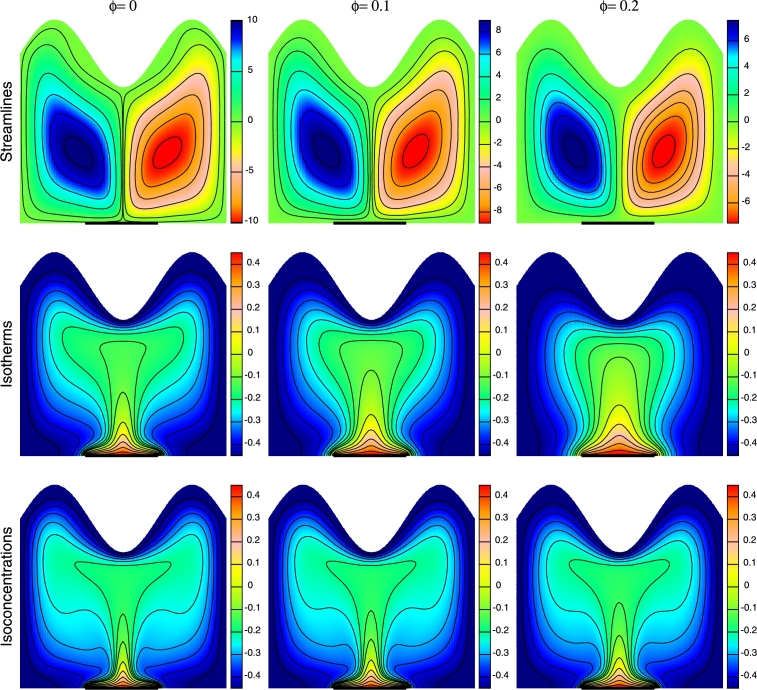
Streamlines, Isotherms and Isoconcentrations for different volume fraction of nanoparticles (*ϕ*) at *Ra* = 10^5^, *Ha* = 30, *n* = 3 and *N* = −2.

Influence of nanoparticle volume fractions on streamlines, isotherms and isoconcentrations is illustrated in Fig. 7, by keeping other parameters constant. The fluid covers the entire enclosure in case of pure fluid (ϕ=0.0). Addition of nanoparticles increases the fluid viscosity and thermal conductivity by increasing the viscous force effect and buoyancy force effect respectively. The strength of flow field attenuates with rise in *ϕ* due to larger viscous effect that slows down the fluid movement. The conductive heat transfer enhances with the addition of nanoparticles in the base fluid. It is noticed that the effect is more pronounced in isotherm and isoconcentration contours as compared to streamlines and they are almost similar near the active part i.e. near the middle part of the bottom wall. Rise in *ϕ* leads to deformation of the thermal and solutal boundary layer at the heated surface and the isotherm and isoconcentration contours become linear. The thickness of the plumes that emerges from the discrete heat source is more for clear water as compared to nanofluid (ϕ=0.2). Moreover, the presence of magnetic field tends to drop the convection effect as the contours become less curved and flattened. Due to high thermal conductivity of nanoparticles, conductive mode of heat transfer dominates.

### 5.5 Effects of undulation number *n*

**Figure 8:**
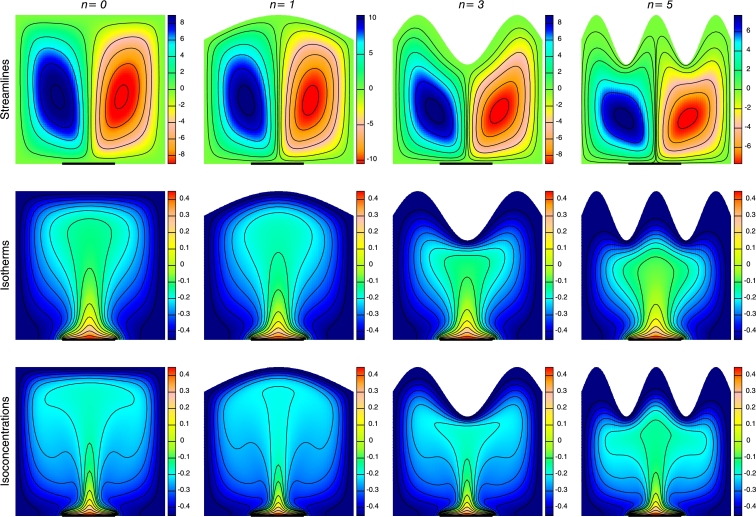
Streamlines, Isotherms and Isoconcentrations for different undulation numbers (*n*) at *Ra* = 10^5^, *Ha* = 30, *N* = −2 and *ϕ* = 0.1.

Fig. 8 exposes the streamlines, isotherms and isoconcentrations for various values of the undulation number n(=0−5) by keeping other parameters fixed. The fluid flow intensity gets weaken and the eye of circulation becomes tilted by increasing the number of waves that leads to intense cooling of the enclosure. Distortion of the streamlines are found due to the presence of wavy wall. It is also observed that the heat rises from the middle portion of the lower wall due to discrete heating of that portion while the rest lower wall are kept adiabatic. Increase in *n* has less significant effect at the core region than at the region adjacent to the upper wall. For n=0 (absence of wave), the isotherm and isoconcentration contours are found to be less bent, whereas with the increase of *n*, the contours form sinusoidal wave near the wavy wall. The isotherm and isoconcentration contours of low temperature are found near the vertical and top wavy wall whereas contours of high temperature are found near the lower wall.

### 5.6 Entropy generation

**Figure 9:**
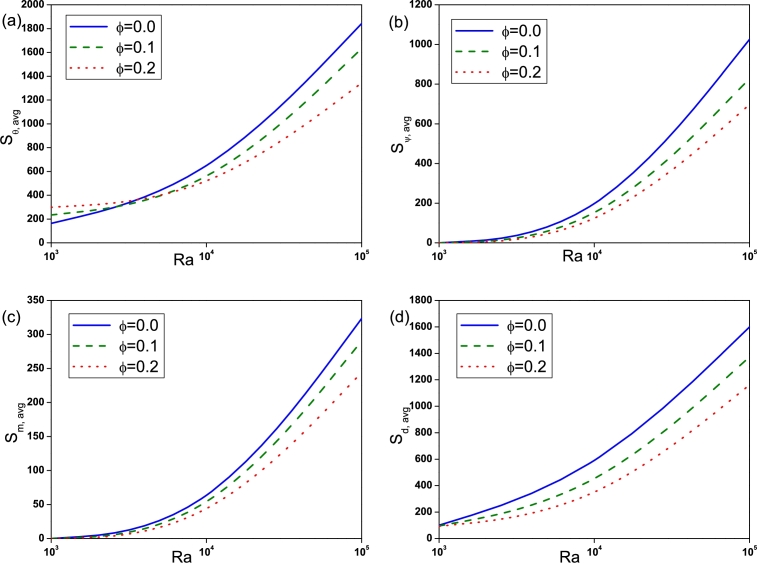
(a) Entropy generation due to heat transfer (*S*_*θ*,*avg*_), (b) entropy generation due to fluid friction (*S*_*ψ*,*avg*_), (c) entropy generation due to magnetic effect (*S*_*m*,*avg*_) and (d) entropy generation due to diffusion (*S*_*d*,*avg*_) for different *Ra* with *Ha* = 30, *N* = −2 and *n* = 3.

**Figure 10:**
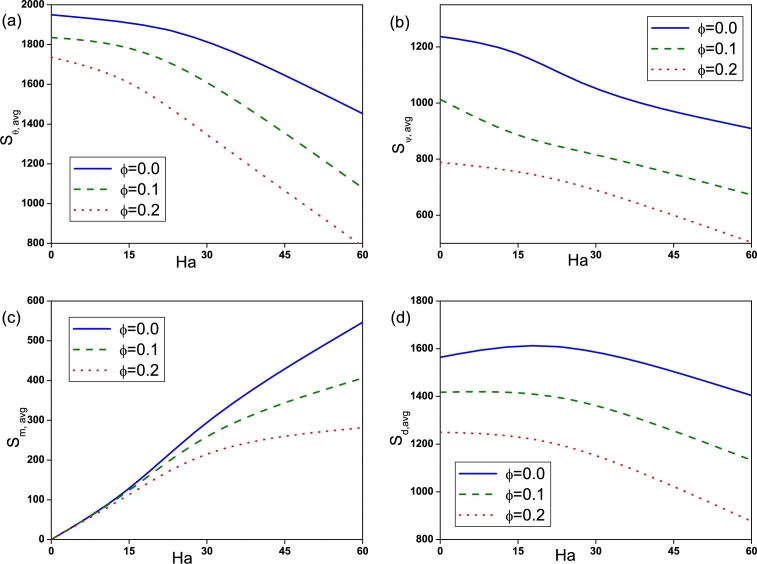
(a) Entropy generation due to heat transfer (*S*_*θ*,*avg*_), (b) entropy generation due to fluid friction (*S*_*ψ*,*avg*_), (c) entropy generation due to magnetic effect (*S*_*m*,*avg*_) and (d) entropy generation due to diffusion (*S*_*d*,*avg*_) for different *Ha* with *Ra* = 10^5^, *N* = −2 and *n* = 3.

**Figure 11:**
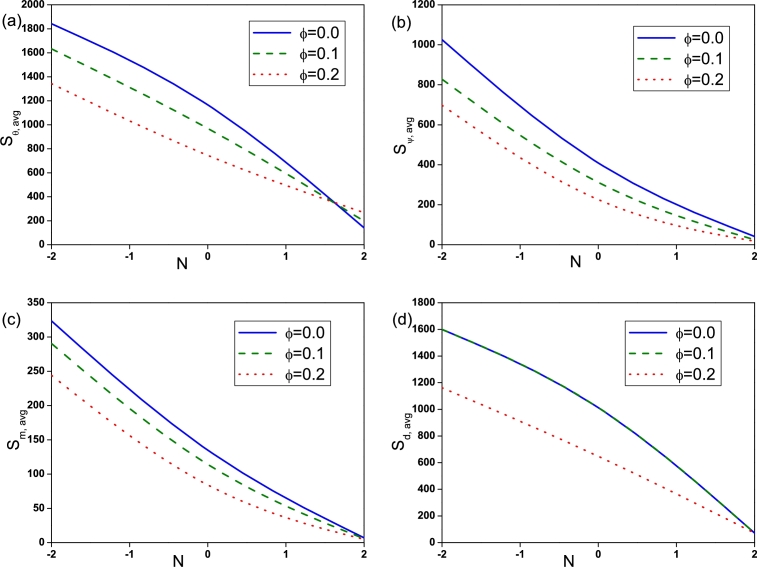
(a) Entropy generation due to heat transfer (*S*_*θ*,*avg*_), (b) entropy generation due to fluid friction (*S*_*ψ*,*avg*_), (c) entropy generation due to magnetic effect (*S*_*m*,*avg*_) and (d) entropy generation due to diffusion (*S*_*d*,*avg*_) for different *N* with *Ra* = 10^5^, *Ha* = 30 and *n* = 3.

**Figure 12:**
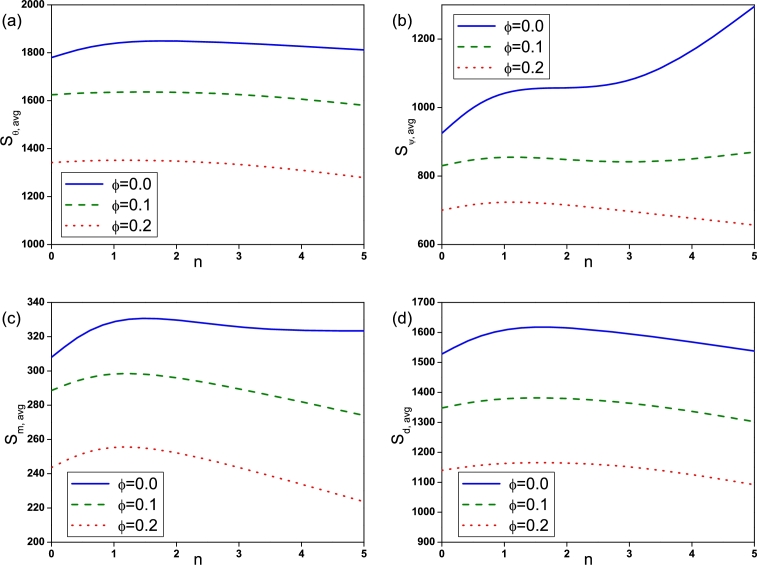
(a) Entropy generation due to heat transfer (*S*_*θ*,*avg*_), (b) entropy generation due to fluid friction (*S*_*ψ*,*avg*_), (c) entropy generation due to magnetic effect (*S*_*m*,*avg*_) and (d) entropy generation due to diffusion (*S*_*d*,*avg*_) for different *n* with *Ra* = 10^5^, *Ha* = 30 and *N* = −2.

**Figure 13:**
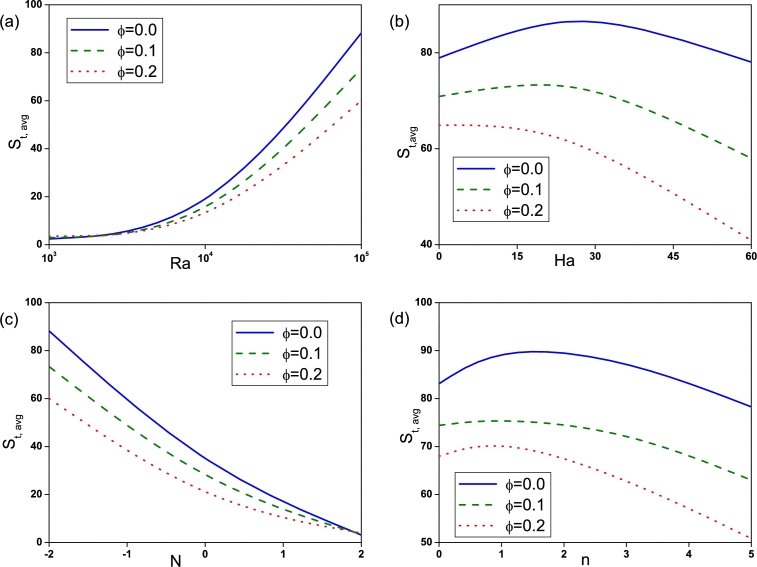
Variation of total entropy generation (*S*_*t*,*avg*_) for different (a) *Ra*, (b) *Ha*, (c) *N* and (d) *n*.

The effect of Rayleigh number on $S_{\theta ,avg}$, $S_{\psi ,avg}$, $S_{m,avg}$ and $S_{d,avg}$ is depicted by Fig. 9. $S_{\theta ,avg}$ for nanofluid (ϕ=0.2) is found to be high at low $Ra=10^{3}$ compared to pure fluid (ϕ=0.0). With the increase of *Ra* entropy generation due to heat transfer, fluid friction, magnetic field and diffusion increases and is found to be high for pure fluid (ϕ=0.0) at high $Ra=10^{5}$. The influence of Hartmann number is displayed in Fig. 10. It is observed that $S_{\theta ,avg}$, $S_{\psi ,avg}$ and $S_{d,avg}$ declines with an augmentation in *Ha* due to the presence of Lorentz force. It is worthy noticed that $S_{m,avg}$ rises with *Ha*. Fig. 11 illustrates the effect of Buoyancy ratio on $S_{\theta ,avg}$, $S_{\psi ,avg}$, $S_{m,avg}$ and $S_{d,avg}$. It is determined that the average entropy generation due to all attenuates with rise in *N* for both pure fluid and nanofluid and found to be higher for pure fluid (ϕ=0.0). Fig. 12 depicts the influence of undulation number on entropy generation. Rise in undulation number causes decrease in entropy generation. Fig. 13 shows the influence of considered parameters on total entropy generation. It is clear from Figure that $S_{t,avg}$ rises with an augmentation in *Ra* and declines with rise in *N*, *Ha* and *n*. Also, $S_{t,avg}$ drops with increase of volume fraction of nanoparticles. Rise in *ϕ* enhances the thermal conductivity that consequently enhances the temperature and concentration gradient that leads to reduction in entropy generation.

### 5.7 Heat and mass transfer rates: average Nusselt and Sherwood numbers

**Figure 14:**
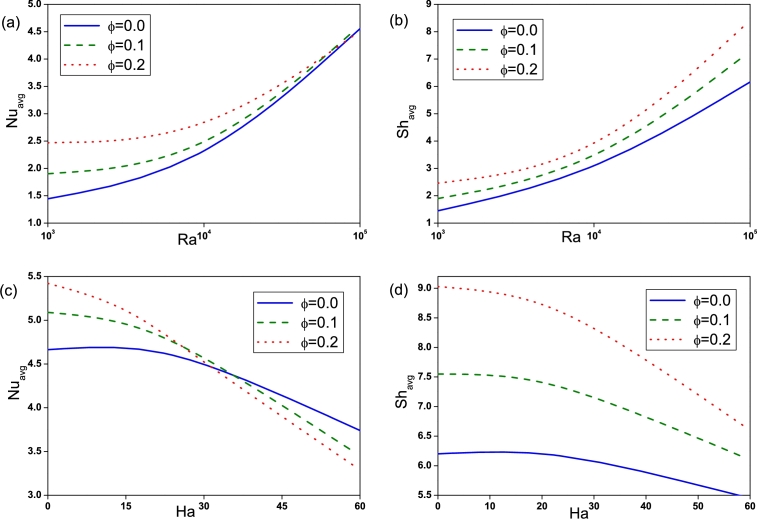
Variation of average Nusselt number and Sherwood number for different *Ra* ((a) and (b)) and *Ha* ((c) and (d)).

**Figure 15:**
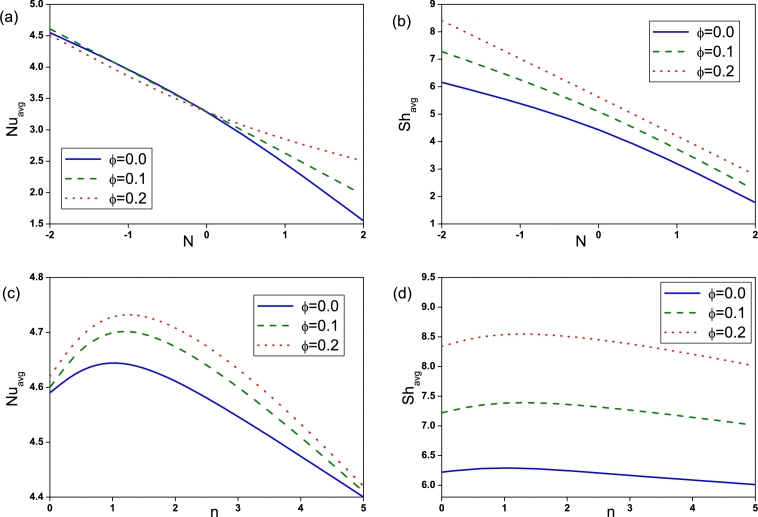
Variation of average Nusselt number and Sherwood number for different *N* ((a) and (b)) and *n* ((c) and (d)).

The heat and mass transfer rate is determined using the average Nusselt and Sherwood numbers respectively. From Fig. 14(a) and (b), it can be seen that irrespective of other parameters, the increase in *Ra* escalates $Nu_{avg}$ and $Sh_{avg}$ due to enhanced convection and consequently heat and mass transfer are increasing function of Rayleigh number. Influence of Hartmann number on Average Nusselt number and Sherwood number is plotted in Fig. 14(c) and (d). $Nu_{avg}$ and $Sh_{avg}$ attenuates with enhancement of *Ha* as the presence of strong magnetic field intensifies the conduction mechanism and consequently reduces the heat and mass transfer rate. The rate of heat and mass transfer are affected by buoyancy ratio in such a way that values of $Nu_{avg}$ and $Sh_{avg}$ at (N=2) are always less than the corresponding values at (N=−2), as presented in Fig. 15(a) and (b). Heat and mass transfer rate decreases with increasing undulation number and the maximum value is attained for n=1, as shown in Fig. 15(c) and (d). It is clear that the addition of nanoparticles in presence of magnetic field improves the heat and mass transfer rate and hence increases the $Nu_{avg}$ and $Sh_{avg}$. The effect is found to be more pronounced in case of low *Ra* than at high *Ra*.

## 6 Conclusions

The present work is done to investigate heat and mass transfer in a wavy enclosure discretely heated and concentrated from the lower wall and filled with nanofluid. The upper and the vertical walls are maintained cold while the remaining part of the lower wall are considered to be adiabatic. This work is analyzed on the basis of various parameters and obtained results can be summarized as follows:

• The flow strength augments with an amplification in *Ra* and declines with rise in buoyancy ratio, Hartmann number, undulation number and volume fraction of nanoparticles.
• Convection mode increases with rise of Rayleigh number but it attenuates with rise of Lorentz forces.
• The behavior of the fluid streamlines, isotherms, and iso-concentrations within the enclosure is found to be strongly dependent upon the considered parameters.
• The average Nusselt number and Sherwood number increases with increase in *Ra* and attenuates with increase in Hartmann number and nanofluid volume fraction.
• Increase in undulation number and buoyancy ratio decreases the performance of heat and mass transfer rate. The fluid flows in reversed direction when *N* is positive resulting in lower heat and mass transfer rate due to the opposing gradients, and fluid rotates in an anticlockwise direction.
• The addition of Al_2_O_3_ nanoparticle leads to attenuation of convective flow and is found to be most effective in enhancing the performance of heat and mass transfer rates.
• The enhancement of Rayleigh number increases the total entropy generation, while the enhancement of buoyancy ratio, undulation number and Hartmann number decreases the total entropy generation.

## Declarations

### Author contribution statement

Rujda Parveen: Conceived and designed the analysis; Analyzed and interpreted the data; Wrote the paper. T.R. Mahapatra: Conceived and designed the analysis; Wrote the paper.

### Funding statement

Rujda Parveen was supported by Department of Science and Technology (DST) INSPIRE (No: DST/INSPIRE Fellowship/[IF170617]), India. T.R. Mahapatra was supported by (SAP-I) Refs.: F.510/3/DRS-III/2015 under UGC, New Delhi, India.

### Competing interest statement

The authors declare no conflict of interest.

### Additional information

No additional information is available for this paper.
